# Proteomic and functional analysis of alpaca (*Vicugna pacos*) sperm quality following *in vitro* capacitation with follicular and oviductal fluids

**DOI:** 10.3389/fvets.2025.1702095

**Published:** 2025-11-06

**Authors:** Edith A. Torres Hualla, Alba Martiarena, María Gabriela Buglio Ballesteros, Maribel Fortunata Medina Rojas, Cristian Rivera Chino, Daniel Gandarillas Espezua, Martin E. Argañaraz

**Affiliations:** 1 Escuela de Medicina Veterinaria y Zootecnia, Universidad Nacional Jorge Basadre Grohmann, Tacna, Peru; 2 Instituto Superior de Investigaciones Biológicas (INSIBIO), Consejo Nacional de Investigaciones Científicas y Técnicas, Universidad Nacional de Tucumán (CONICET-UNT), Instituto de Biología “Dr. Francisco D. Barbieri”, Facultad de Bioquímica, Química y Farmacia, UNT, San Miguel de Tucumán, Argentina; 3 Cátedra de Biología Celular y Molecular, Facultad de Bioquímica, Química y Farmacia, Universidad Nacional de Tucumán, San Miguel de Tucumán, Argentina; 4 Laboratorio de Biotecnología Reproductiva Animal, Universidad Nacional Jorge Basadre Grohmann, Tacna, Peru

**Keywords:** alpaca, sperm quality, spermatozoa, follicular fluid, oviductal fluid, sperm capacitation, proteomics

## Abstract

Assisted reproductive technologies, such as *in vitro* fertilization, remain inefficient in camelids, largely due to gaps in understanding the molecular interactions that regulate sperm capacitation. Fertilization requires not only viable spermatozoa but also the precise modulation of capacitation by the peri-ovulatory microenvironment, including follicular fluid (FF) and oviductal fluid (OF). In this study, spermatozoa were incubated in Fert-TALP medium supplemented with FF or OF, and both functional outcomes and proteomic remodeling were assessed. Sperm treatments were evaluated in five independent biological replicates per individual (three individuals), with triplicate proteomics performed. FF (*n* = 20) was collected from pre-ovulatory follicles (7–9 mm) and OF (*n* = 10) from the corresponding ipsilateral oviducts, thereby reflecting the *in vivo* environment encountered by sperm in the female reproductive tract following mating. Incubation with FF enhanced progressive motility by 72%, rapid progressive motility by 169%, viability by 30%, and acrosome responsiveness by 30%, and was associated with a proteomic shift involving ~12% of proteins (*p* < 0.05). These included factors implicated in zona pellucida binding (LYPD4, PGK1, ANXA2, and TCP1 complex members) and galactose metabolism (MAOA, AKR1B1, GLA, and HK1). The enriched processes included glycolysis/gluconeogenesis, cytoskeletal reorganization, and protein maturation, all consistent with sperm capacitation. By contrast, sperm incubated with OF showed an underrepresentation of capacitation-related pathways, including the proteasome complex, sperm fibrous sheath, and TCA cycle. Moreover, the OF proteome (*r* = 2) revealed decapacitation-associated factors such as PEBP1 and PAFAH1B3, which likely stabilize membranes and delay premature capacitation. Together, these findings demonstrate complementary yet contrasting roles of FF and OF in modulating sperm physiology: FF acting as a capacitating medium, and OF providing a stabilizing environment. This study presents the first partial proteome of capacitated alpaca sperm together with matched reproductive fluids, providing mechanistic insights with direct implications for improving assisted reproduction in camelids.

## 1 Introduction

During the application of assisted reproductive technologies, ensuring the preservation and enhancement of sperm quality is essential for achieving optimal fertilization rates and successful embryo development (1). Sperm quality is typically evaluated based on a set of viability parameters, including motility, membrane integrity, acrosomal status, and overall viability. To support these parameters *in vitro*, capacitation media are formulated to simulate the biochemical and physiological conditions encountered by spermatozoa within the female reproductive tract. However, accurately replicating this dynamic environment remains a significant challenge. The composition of uterine and oviductal fluids is not static; rather, it varies both temporally and spatially with the stage of the estrus or ovulatory cycle (2). Moreover, fertilization in mammals occurs within the oviduct, where both spermatozoa and the ovulated oocyte converge in a highly specialized microenvironment. During ovulation, a small but physiologically relevant proportion of follicular fluid (approximately 0.5%) is released into the oviduct along with the oocyte, thereby modifying the local biochemical milieu and adding further complexity to the fertilization environment (3). Notably, follicular fluid (FF) has been recognized as a key modulator of sperm function, enhancing capacitation and promoting the acrosome reaction in various mammalian species (4).

Indeed, it has been reported that supplementing capacitation media with FF or OF improves sperm quality during *in vitro* fertilization. This has been described in several species, such as bulls (5, 6), rams (7), equines (8, 9), and boars (10, 11). These fluids interact with spermatozoa *via* peptides, hormones, and metabolites, enhancing oocyte recognition and facilitating the acrosome reaction during fertilization (12, 13).

Although the biochemical composition of follicular fluid (FF) and oviductal fluid (OF)—including hormones, proteins, and phospholipids—has been described in various species (14–16), the specific proteins involved in sperm capacitation and their influence on sperm quality parameters remain unclear. These observations underscore the importance of understanding how native reproductive tract fluids influence sperm physiology and highlight the need to explore their potential as functional supplements in capacitation media designed for *in vitro* applications.

The reproductive physiology of South American camelids (SAC) is not yet fully understood, which limits progress in developing effective artificial insemination (AI) techniques. Unlike cattle, where AI is routinely implemented, the application of AI in SAC is constrained by uncertainties regarding the optimal timing relative to ovulation, appropriate sperm dosage, deposition site, and unresolved issues in semen preservation. SAC are induced ovulators, with ovulation triggered by nerve growth factor (NGF) in seminal plasma. As a result, ovulation induction is frequently necessary during AI procedures, although the ideal interval between induction and semen deposition remains undefined. Additional challenges include low sperm concentration, high ejaculate viscosity, and thread formation (17).

Female SAC exhibit distinct reproductive characteristics, including a short corpus luteum lifespan, asymmetrical luteolytic activity between uterine horns, a brief period for maternal recognition of pregnancy (MRP), and implantation limited to the left uterine horn (18). These features, together with induced ovulation, indicate that reproductive knowledge from spontaneous ovulators, such as cattle or pigs, cannot be directly transferred to camelids (17, 18). Therefore, enhancing AI efficiency in SAC necessitates a comprehensive understanding of their unique reproductive physiology, particularly the interactions between sperm and the peri-ovulatory microenvironment.

*In vitro* studies of sperm capacitation using native FF and OF provide a valuable model for replicating the peri-ovulatory microenvironment observed *in vivo*. Such investigations are essential for clarifying alpaca reproductive physiology and informing the development of species-specific AI protocols.

In alpacas, both the protein composition and the effects of FF and OF on sperm quality remain poorly characterized. Proteomic analysis offers a powerful approach to elucidate protein interactions and their roles in sperm function (19, 20). Therefore, the objectives of this study were to evaluate the quality of alpaca spermatozoa (*Vicugna pacos*) following *in vitro* capacitation using media supplemented with 20% FF or OF and to characterize the proteomic interactions between these fluids and sperm during the capacitation process.

## 2 Materials and methods

### 2.1 Study site

This study was conducted at the Animal Reproductive Biotechnology Laboratory of the Universidad Nacional Jorge Basadre Grohmann (UNJBG) in Tacna, Peru, located at UTM coordinates 366907, 8006025, and at an average altitude of 599 m above sea level. The local climate in this region ranges from 22 °C to 30 °C during the summer and from 10 °C to 21 °C during the winter. This study adhered to the ethical guidelines established by the Ethics Committee of the UNJBG (ethics approval code: 2023-024-CEIUNJBG).

### 2.2 Sample collection and preparation

Follicular and oviductal fluids were obtained from female alpaca reproductive tracts collected at the Masocruz slaughterhouse in Puno, approximately 8 h from Tacna. Samples were transported in a refrigerated container of 0.9% NaCl at 5 °C. Semen collection procedures were conducted at the Animal Reproductive Biotechnology Laboratory of UNJBG.

#### 2.2.1 Follicular fluid (FF)

Upon arrival at the laboratory, mature pre-ovulatory ovarian follicles measuring 7–9 mm in diameter (*n* = 20) were aspirated using a 21G needle. The collected FF was centrifuged at 5,000 rpm for 15 min, and this step was repeated three times to ensure the complete removal of cellular components. The resulting supernatant was filtered through a 0.45-μm membrane and stored at −20 °C until use for sperm capacitation experiments and proteomic analysis by mass spectrometry (MS).

#### 2.2.2 Oviductal fluid (OF)

The ipsilateral oviducts (*n* = 10) of ovaries bearing 7–9 mm pre-ovulatory follicles were used. Each oviduct was flushed in a retrograde direction using a 1-ml syringe fitted with a 25G needle containing 200 μL of 0.9% NaCl. Gentle pressure was applied along the length of the oviduct using a hemostatic clamp to facilitate fluid recovery, which was collected into 500 μL Eppendorf tubes (21, 22). The recovery success rate was approximately 80%. The collected fluid was centrifuged at 5,000 rpm for 15 min, a step that was repeated two times, followed by filtration through a 0.45-μm membrane. The supernatant was then stored at −20 °C. A portion of the OF sample was used for proteomic analysis by mass spectrometry (MS).

#### 2.2.3 Semen collection and sample preparation

Fifteen ejaculates were collected with an artificial vagina and mannequin (23) from three adult male alpacas (five ejaculates per individual, aged 5–8 years). Only ejaculates meeting minimum sperm quality parameters (total motility >50%, sperm concentration >70 × 10^6^/ml, and viability >50%) were included (23, 24). Ejaculates were pooled, centrifuged at 3,500 rpm for 5 min to remove seminal plasma, and the sperm pellet was resuspended in Fert-TALP medium. The pooled sample was then divided into three treatment groups: control (Fert-TALP only), FF (Fert-TALP + 20% follicular fluid), and OF (Fert-TALP + 20% oviductal fluid).

### 2.3 *In vitro* sperm capacitation

Capacitation was performed using Fertilization Tyrode’s Albumin Lactate Pyruvate (Fert-TALP) medium (25, 26), which contained 114 mM NaCl, 3.2 mM KCl, 25 mM NaHCO₃, 0.4 mM NaH₂PO₄, 2 mM CaCl₂·2H₂O, 0.5 mM MgCl₂·6H₂O, 10 mM sodium lactate, 10 mM sodium pyruvate, 6 mg/mL fatty acid-free BSA, 50 μg/mL streptomycin, and 1 μL/mL phenol red. The medium was freshly prepared on the day of use and equilibrated for 4 h at 38.8 °C in 5% CO₂ to achieve an osmolarity of 360 mOsm and a pH of 7.45. Sperm pellets obtained after centrifugation were resuspended in 100 μL of Fert-TALP, and concentrations were determined using a Neubauer chamber (Sigma–Aldrich, United States). For each treatment, aliquots containing 20 × 10^6^ spermatozoa were adjusted to a final volume of 500 μL. Samples were incubated for 3 h at 38.8 °C in an atmosphere of 5% CO₂ and 100% humidity. Following incubation, sperm functional parameters, including motility kinetics, functional membrane integrity (HOST), acrosome status (FITC-PSA), and viability (Hoechst 33342/PI staining), were assessed. Each treatment was tested in five independent biological replicates (*n* = 5). For proteomic analysis, an additional set of samples from the same pooled ejaculates was processed in triplicate for each treatment (*n* = 3) and analyzed by LC–MS/MS.

### 2.4 Evaluation of sperm parameters

#### 2.4.1 Sperm kinetics

Three microliters (3 μL) of diluted semen from each treatment group were placed on a Leja chamber (Androvision, Minitube, United States) and analyzed using the computer-assisted sperm analysis system (CASA; Androvision, Minitube, United States). The “sperm motility” program was selected, and five different fields of view were evaluated to determine total motility, progressive motility, and immotile sperm. The CASA system also recorded kinetic parameters, including curvilinear distance (DCL, μm), straight-line distance (DSL, μm), curvilinear velocity (VCL, μm/s), straight-line velocity (VSL, μm/s), linearity (VSL/VCL), and amplitude of lateral head displacement (ALH).

#### 2.4.2 Sperm membrane functional integrity (HOST)

**Figure 1:**
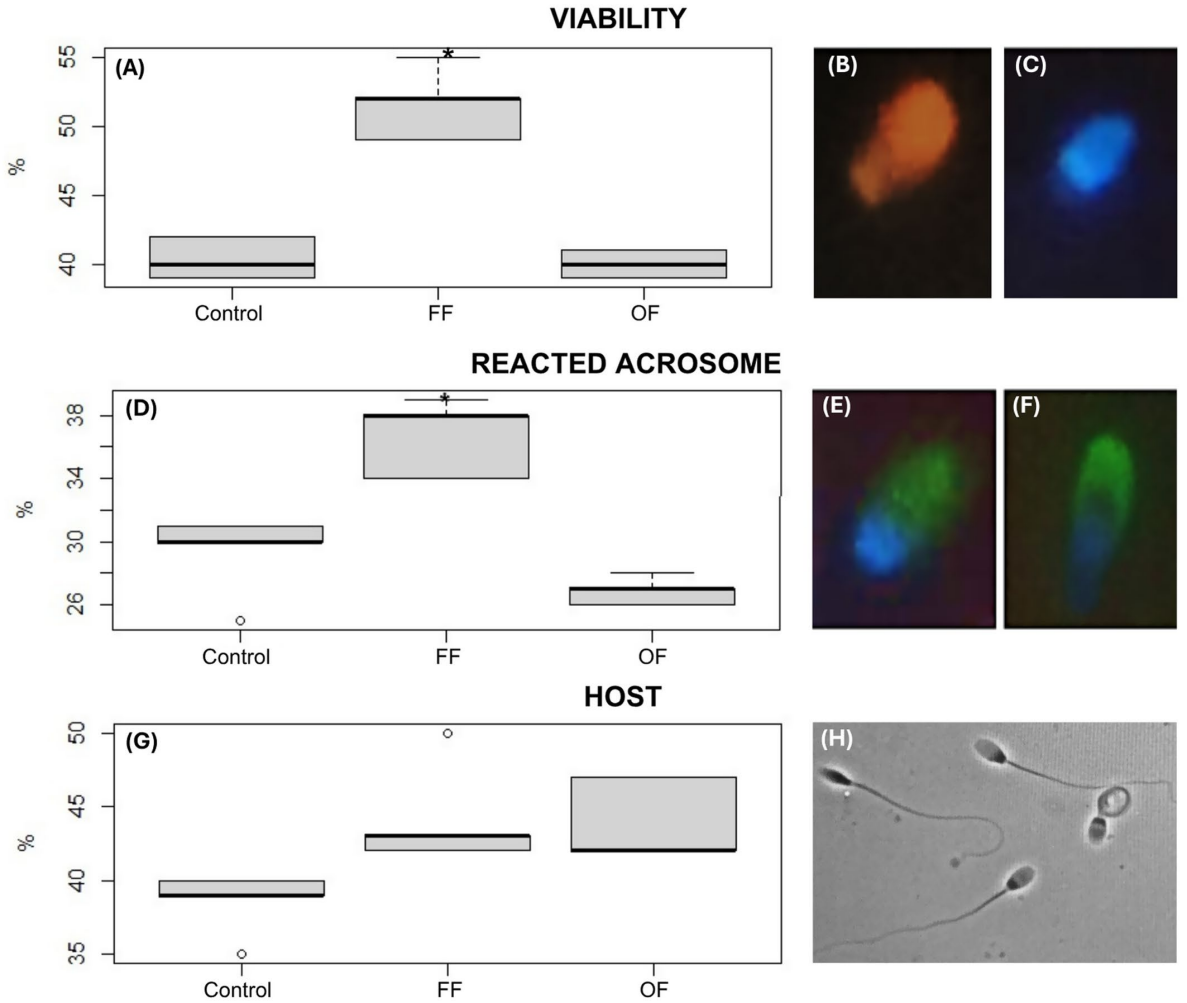
Functional parameters of alpaca spermatozoa after *in vitro* capacitation. Boxplots show sperm viability **(A)**, acrosome reaction **(D)**, and membrane integrity assessed by the hypoosmotic swelling test (HOST) **(G)** after 3 h of incubation in Fert-TALP medium under different treatments: Control, follicular fluid (FF), and oviductal fluid (OF). Significant differences are indicated (* *p* < 0.05). Representative fluorescence images illustrate sperm viability using Hoechst 33342 and propidium iodide (P4170): non-viable cells with red/fuchsia heads **(B)** and viable cells with blue heads **(C)**. Acrosome status was evaluated with FITC-PSA: intact acrosome **(E**, continuous green halo**)** and reacted acrosome **(F**, discontinuous green signal**)**. HOST was performed by assessing sperm response to a hypoosmotic solution under an optical microscope at 400× magnification, with sperm showing tail swelling or coiling considered reacted **(H)**. FF-treated sperm displayed higher viability and acrosome reaction compared with Control and OF groups, with a trend toward improved membrane integrity.

The hypoosmotic solution (150 mOsm) was prepared with fructose (13.51 g) and sodium citrate dihydrate (7.35 g) in 1 L of distilled water (27). A vial containing 90 μL of the hypoosmotic solution was pre-warmed in a water bath at 37 °C. Ten μl of semen was added and incubated for 30 min. After incubation, 20 μL of 5% formalin was added, and the sample was removed from the bath. Sperm response to the hypoosmotic solution was evaluated under an optical microscope at 400X magnification. Spermatozoa exhibiting tail swelling or coiling were considered to have reacted, and the percentage was calculated from 200 cells (Figure 1H).

#### 2.4.3 Reacted acrosome: FITC-PSA fluorescent staining (Pisum sativa)

To evaluate acrosomal integrity, sperm samples were first permeabilized with 4% paraformaldehyde (Sigma–Aldrich, P-6148) on a microscope slide for 5 min and then washed two times with 1x PBS. For acrosome status assessment, spermatozoa were incubated with FITC-PSA (Sigma–Aldrich, L0770) for 30 min and examined under a fluorescence microscope (Olympus IX73). Sperm displaying fluorescence over the anterior half of the head were considered to have an intact acrosome (Figure 1E). When fluorescence was partially present or scattered, the acrosome was considered reacted (1) (Figure 1F).

#### 2.4.4 Sperm viability: Hoechst and propidium iodide staining

An aliquot of 3 μL of semen was placed in a dark vial, followed by 1.5 μL of Hoechst (1 mM; Sigma–Aldrich, 33342, United States) and 1 μL of propidium iodide (0.5 mM; Sigma–Aldrich, P4170, United States). The mixture was incubated at 37 °C for 5 min (28). After incubation, 3 μL of the stained sample was placed on a slide and analyzed using the sperm viability module of the CASA system. Sperm stained blue were considered viable (Figure 1C), whereas sperm stained pink-red were considered non-viable (Figure 1B). A minimum of 500 spermatozoa were evaluated to determine the percentage of viable and non-viable cells.

### 2.5 SDS–PAGE, in-gel digestion and LC–MS/MS

Twenty micrograms of total protein was loaded per sample and separated on a 1-cm segment of a 10% SDS–PAGE gel at 150 V. Samples included *in vitro*–capacitated spermatozoa from each treatment group (control, FF, and OF; *n* = 3 per group), as well as follicular fluid and oviductal fluid samples. A single pooled sample, prepared as described above (Section 2.2), was analyzed in duplicate (*r* = 2) for each fluid type. Following electrophoresis, the gels were incubated in a fixing solution containing 30% (v/v) ethanol and 2% H₃PO₄ for 3 h. Subsequently, they were immersed in a staining solution containing 18% (v/v) methanol, 17% (w/v) (NH₄)₂SO₄, and 2% (v/v) H₃PO₄ under continuous shaking for 1 h. Coomassie G-250 powder (0.5 g/L) was added to the staining solution, and the gels were incubated for an additional 2 days to visualize protein bands. Each lane was cut using a scalpel and sent to the Proteomics Core Facility of CEQUIBIEM (Centro de Estudios Químicos y Biológicos por Espectrometría de Masa), University of Buenos Aires, Argentina, where protein digestion and mass spectrometry analysis were performed separately (29).

There, samples were reduced with 20 mM DTT for 45 min at 56 °C and alkylated with 50 mM iodoacetamide for 45 min in the dark. They were then digested with molecular-grade trypsin overnight. Peptides were extracted using acetonitrile. Samples were lyophilized using a Speed Vac and resuspended in 30 μL of 0.1% trifluoroacetic acid. Desalting was performed using Zip-Tip C18. A gradient formed by mobile phase A (water, 0.1% formic acid) and mobile phase B (acetonitrile, 0.1% formic acid) was used to separate the sample content at a 0.3-μl/min flow rate. The following gradient elution was used for peptide separation: 7–30% of mobile phase B in 60 min, 30–95% of mobile phase B in 2 min, and held at 95% of mobile phase B for 13 min. Proteins were analyzed by nanoHPLC (EASY-nLC 1,000, Thermo Scientific, Germany) coupled to a mass spectrometer with Orbitrap technology (Q-Exactive with High Collision Dissociation cell and Orbitrap analyzer, Thermo Scientific, Germany). Peptide ionization was performed by electrospray. The parameters used during the mass spectrometry analysis were as follows: Full MS range: 400–2000; Full MS resolution: 70,000; MS/MS resolution: 17,500.

The mass spectrometer’s raw files were analyzed using the Proteome Discoverer 1.4 and 2.2 software (Thermo Scientific, Germany). Protein identification was performed using the *Vicugna pacos* reference proteome (UniProt Proteome ID: UP000504605). Proteome Discoverer searches were performed with a precursor-mass tolerance of 10 ppm and product-ion tolerance of 0.05 Da. Static modifications were set to carbamidomethylation of cysteine, and dynamic modifications were set to oxidation of Met and N-terminal acetylation. Protein hits were filtered for high-confidence peptide matches with a maximum protein and peptide false discovery rate of 1% calculated using a reverse database strategy. Only proteins with at least two detected peptides were considered. The identified peptides were searched against the *Homo sapiens* database to exclude human contaminants. Proteome Discoverer calculated an area for each protein in each condition using the area under the curve of the three most intense peptides per protein. Areas were calculated for each of the three triplicates and normalized. The mass spectrometry proteomics data have been deposited to the ProteomeXchange Consortium via the PRIDE partner repository with the dataset identifier PXD068210 (https://www.ebi.ac.uk/pride/archive/projects/PXD068210).

### 2.6 Bioinformatics analysis

The data obtained were processed with the Perseus program (Max Planck Institute of Biochemistry, 1.5.5.3 version, freely available online), which allows a more detailed statistical analysis. Different scatter plots were generated according to the samples compared. We plotted the -log10 (Student’s *t*-test *p*-value, A_B) on the y-axis *versus* the Student’s *t*-test difference (A_B) on the x-axis for each pair of samples. Proteins that appeared in the volcano plot with a fold change greater than 2 (less than −1 or greater than 1 on the x-axis) and a *p*-value below 0.05 (above 1.3 on the y-axis) were considered differentially expressed.

Functional enrichment analysis was performed using the Metascape online tool (version 3.5) (30). The identified alpaca sperm proteins were converted to human homologs using BLAST+ (31), since *Vicugna pacos* taxonomy was not available in Metascape. Metascape collected terms with a minimum count of 3, a *p* < 0.01, and an enrichment factor > 1.5 and then grouped them into clusters based on similarities. In the histograms, the most statistically significant term was selected to represent the cluster. The Search Tool for the Retrieval of Interacting Genes/Proteins (STRING, version 11.5) (32) was used for *in silico* analysis based on physical or functional associations, integrating evidence from different sources.

The identified alpaca sperm, follicular fluid, and oviductal fluid proteins were converted to human homologs using BLAST+ (31). For the functional annotation of FF and OF proteins based on KEGG pathways, the online tool Proteomaps (33) was used. To create a Proteomap, the total area was first divided into polygons representing the top-level functional categories. These polygons were constructed from a Voronoi diagram, where the polygons’ areas were defined to represent copy numbers weighted by protein chain lengths (the investment in terms of amino acids, also termed the mass fraction). The top-level areas were then subdivided into subcategories, and the procedure was repeated down to the level of individual proteins. In the Proteomaps, functionally related proteins are arranged in common regions with similar colors (34).

### 2.7 Statistical analysis

Data were analyzed using the statistical software RStudio (version 4.4.2). The results are presented as percentages (%) and expressed as mean ± SEM for each group. The Shapiro–Wilk test was used to assess data normality. Statistical differences between the Control group and the FF and OF treatments were evaluated using one-way ANOVA, followed by Tukey’s *post hoc* test. Non-normally distributed data were analyzed using the Kruskal–Wallis rank test. The results were statistically significant at * *p* < 0.05 and ** *p* < 0.01.

## 3 Results

### 3.1 Effect of follicular and oviductal fluids on sperm motility and kinetic parameters after *in vitro* capacitation

**Figure 2:**
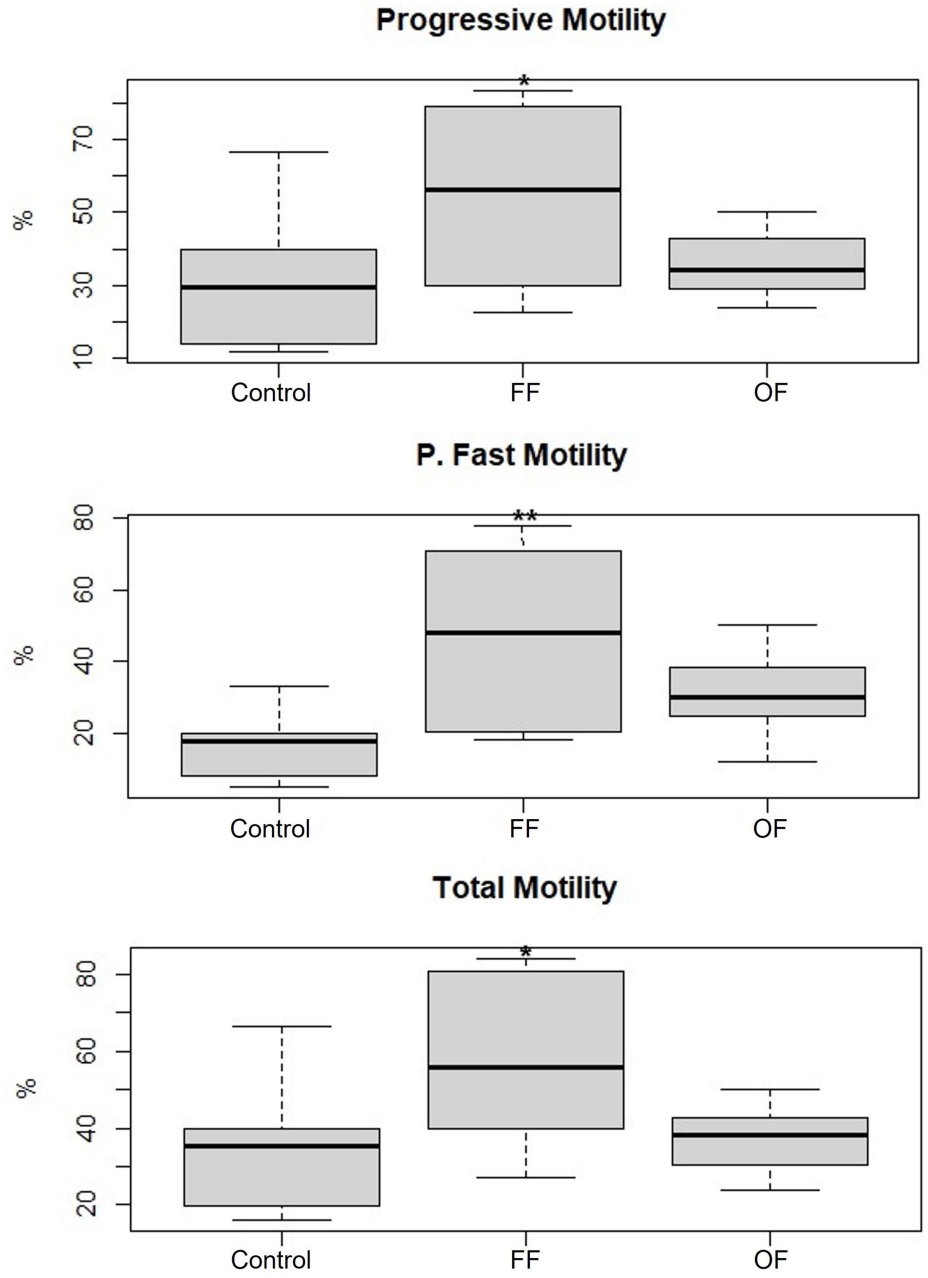
Progressive motility, rapid progressive motility, and total motility of spermatozoa incubated for 3 h in Fert-TALP (Control), Fert-TALP with 20% follicular fluid (FF), and Fert-TALP with 20% oviductal fluid (OF). The data represent mean values, highlighting statistically significant differences in the FF treatment compared with the Control and OF in all three motility parameters (* *p* < 0.05, ** *p* < 0.01).

The sperm motility analysis revealed significant differences among treatments: Control, FF, and OF (Figure 2). Spermatozoa incubated with follicular fluid exhibited a marked improvement in motility parameters. Progressive motility was significantly higher in the FF group (56.84 ± 24.75%) compared with the Control (33.11 ± 21.96%; *p* < 0.05). Similarly, total motility increased significantly in the FF group (59.62 ± 22.61%) compared with the Control group (36.48 ± 19.33%; *p* < 0.05). Notably, rapid progressive motility was also significantly elevated in the FF group (45.85 ± 23.87%) compared with the Control group (17.04 ± 10.82%; *p* < 0.01). In contrast, spermatozoa incubated with oviductal fluid did not show statistically significant differences in any motility parameters when compared with the Control. Progressive motility in the OF group was 36.04 ± 9.39%, rapid progressive motility was 30.54 ± 11.73%, and total motility was 37.27 ± 8.72%.

Analysis of sperm kinetic parameters after 3 h of *in vitro* capacitation in Fert-TALP revealed significant differences in DCL and VCL among treatments (Table 1). Spermatozoa incubated with FF showed a statistically significant increase in both DCL and VCL compared with Control and OF treatments, whereas OF-treated sperm displayed reduced values. No significant differences were detected for VSL or linearity, although a slight, non-significant increase in linearity was noted in the OF group. These results indicate that FF enhances sperm motility, while OF exerts a modest influence on motility patterns.

**Table 1:** Kinetics of progressively motile spermatozoa after *in vitro* capacitation.

Treatment	DCL (μm)	DSL (μm)	VCL (μm/s)	VSL (μm/s)	Linearity
Control	38.53 ± 11.55	7.53 ± 1.64	97.76 ± 6.07*	31.02 ± 11.85	0.26 ± 0.10
FF	47.76 ± 4.09*	10.46 ± 0.72	104.61 ± 7.56**	28.87 ± 2.98	0.28 ± 0.01
OF	33.52 ± 2.58	10.21 ± 1.33	87.45 ± 4.25	27.45 ± 4.05	0.31 ± 0.03

* *p* < 0.05, ** *p* < 0.01.

### 3.2 Sperm functional parameters after *in vitro* capacitation

Post-capacitation evaluation of sperm functional parameters revealed differential effects among the experimental groups, particularly in acrosome reaction and viability (Figure 1). Spermatozoa incubated with FF exhibited a significantly higher percentage of acrosome-reacted cells (37.2 ± 2.65%) compared with the Control (30.45 ± 3.21%; *p* < 0.05). This increase suggests enhanced acrosomal responsiveness in the presence of FF. In contrast, the OF group showed no improvement in this parameter (27.51 ± 10%), with high variability and values comparable to the Control and FF group (Figure 1D).

Regarding membrane functionality, assessed by the HOST test, the FF group showed higher values (45 ± 4.35%) compared with the Control (38 ± 2.65%), and the OF group also demonstrated a modest increase (43.67 ± 2.89%). However, these differences were not statistically significant. As shown in Figure 1G, the FF group displayed a higher median and a wider interquartile range, suggesting a potential stimulatory effect on membrane integrity despite the lack of significance.

Sperm viability was also notably enhanced by follicular fluid. The FF group showed the highest viability (52.7 ± 3.1%), which was significantly greater than the Control and OF groups (*p* < 0.05), as reflected in the pronounced elevation of both median and spread (Figure 1A). In contrast, the OF group showed viability values (40.3 ± 1.0%) similar to the Control.

### 3.3 Multivariate analysis reveals distinct proteomic signatures induced by follicular and oviductal fluids during sperm *in vitro* capacitation

**Figure 3:**
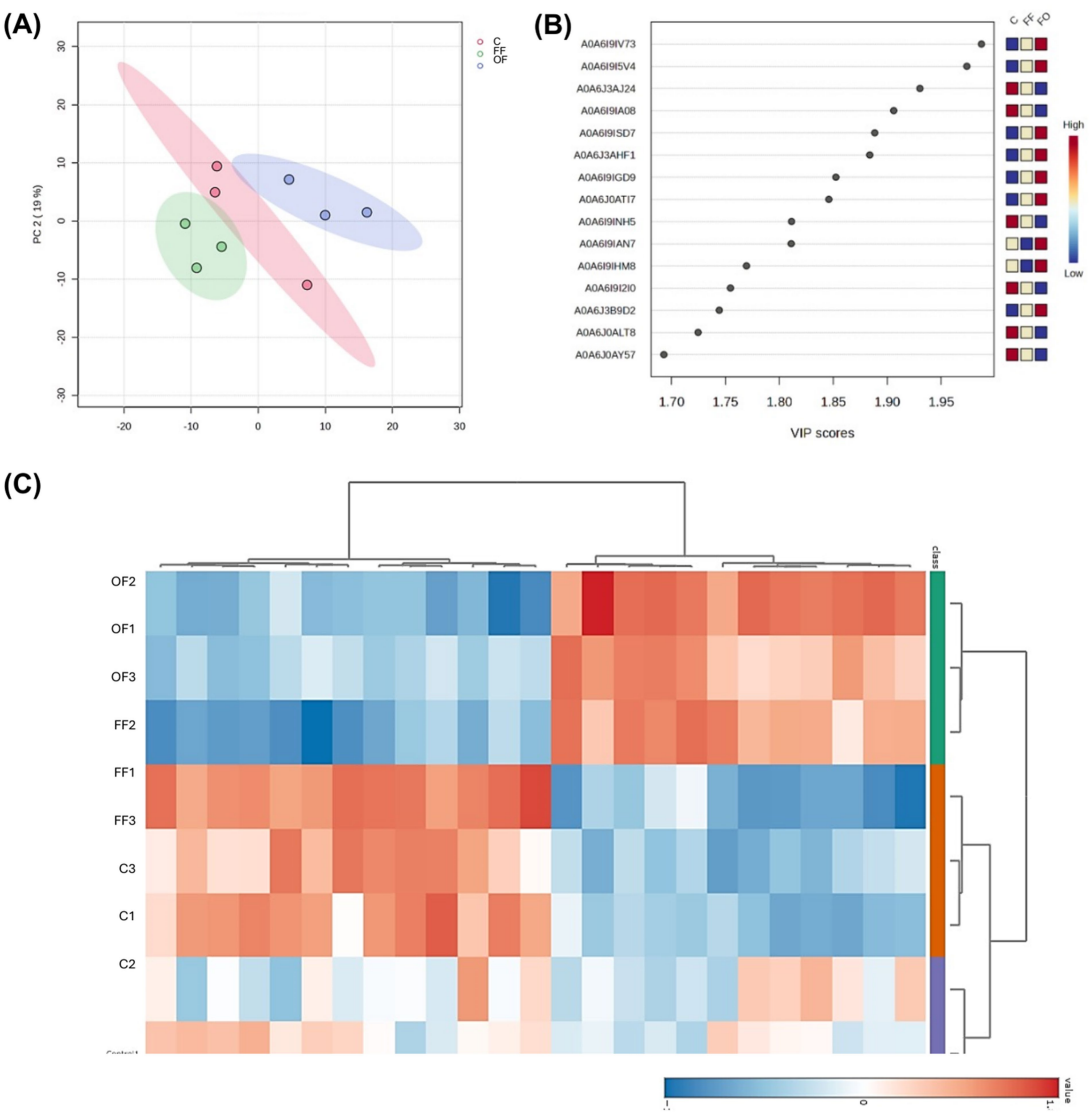
Multivariate analysis of proteomic profiles of spermatozoa incubated with follicular fluid (FF), oviductal fluid (OF), or Control medium (C). **(A)** Principal component analysis (PCA) scores plot showing distinct clustering of FF (red), OF (green), and C (blue) groups (PERMANOVA: *F* = 3.8851, *R*^2^ = 0.56248, *p* = 0.034, 999 permutations). **(B)** Variable importance in projection (VIP) scores plot for the 15 proteins with the highest scores obtained by PLS-DA, with color coding representing relative abundance in each treatment group. **(C)** Heatmap of the 25 proteins with the highest VIP scores, clustered according to Pearson correlation, illustrating differential abundance patterns across treatments.

Multivariate analysis revealed clear proteomic differences among treatments. The PCA plot (Figure 3A) showed distinct clustering of FF, OF, and Control sperm, with the first two components explaining 38.9% of the variance. PERMANOVA confirmed significant differences among groups (*F* = 3.89, *R*^2^ = 0.56, *p* = 0.034). A hierarchical clustering heatmap of the top 25 VIP proteins (Figure 3C) further separated samples by treatment: FF sperm clustered tightly with a characteristic enrichment of proteins related to ATP-dependent activity (GO:0140657), catalytic activity (GO:0003824), and binding (GO:0005488), whereas OF sperm showed reduced abundance of these proteins but higher levels of others. Control samples displayed an intermediate profile, partially overlapping with both FF and OF. PLS-DA identified a set of proteins with high discriminatory power, as indicated by the 15 highest VIP scores (Figure 3B).

### 3.4 Follicular fluid induces significant proteomic remodeling in alpaca spermatozoa

**Figure 4:**
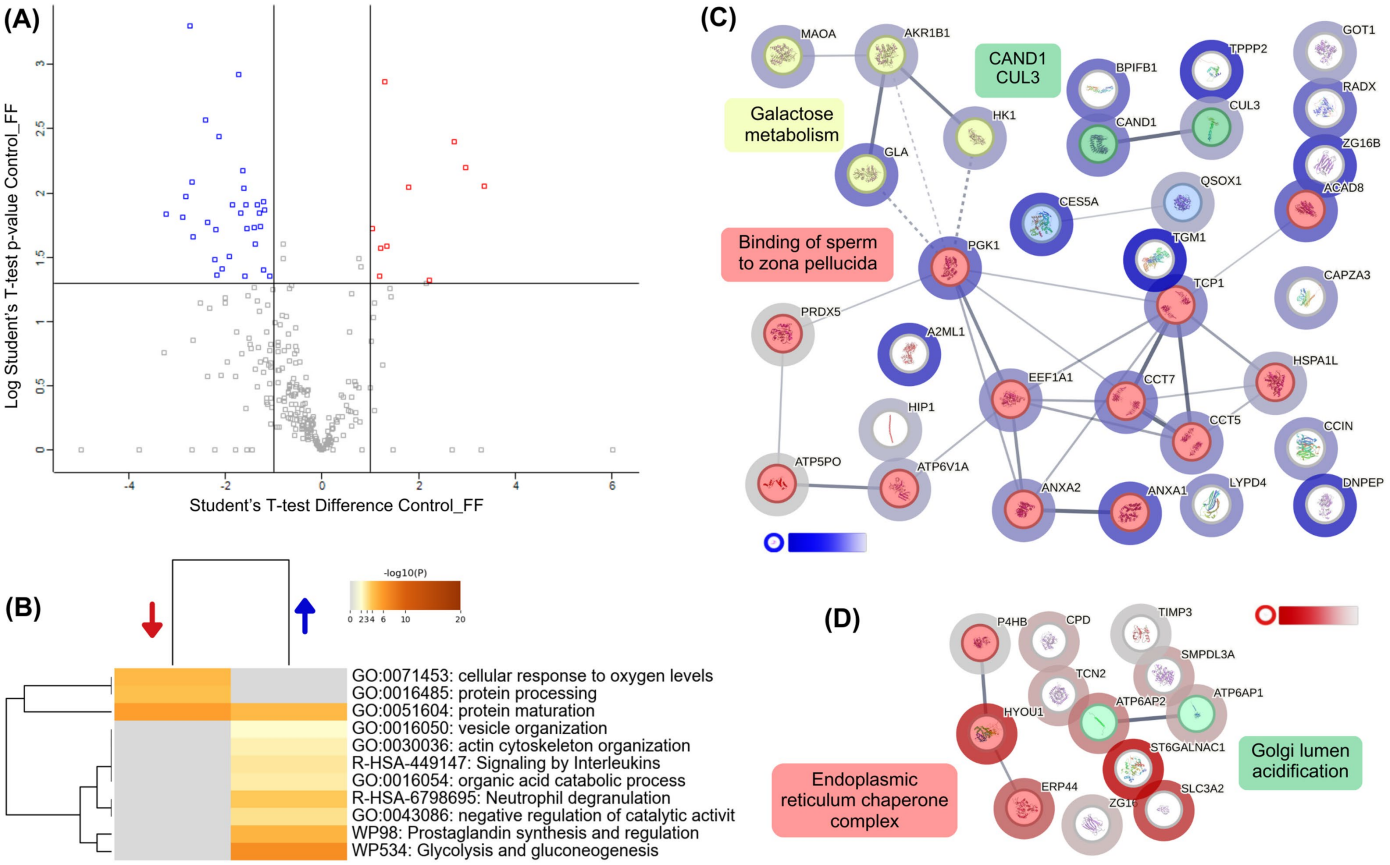
Differential proteomic profile of alpaca spermatozoa incubated with follicular fluid (FF). **(A)** Volcano plot showing proteins differentially abundant between Control (Fert-TALP) and FF-treated spermatozoa. Blue dots indicate proteins overabundant in FF-treated sperm, while red dots represent proteins underabundant (|FC| ≥ 2, *p* < 0.05). **(B)** Heatmap of enriched biological processes identified by Metascape. Blue and red arrows indicate processes associated with overabundant and underabundant proteins, respectively. The representative biological processes include biological processes such as glycolysis/gluconeogenesis, zona pellucida binding, actin cytoskeleton organization, and protein folding. Color intensity corresponds to –log₁₀(*p*-value). **(C)** Protein–protein interaction (PPI) network generated by STRING, clustered using k-means. Nodes are colored according to cluster identity, with halo intensity reflecting the degree of differential abundance: blue halos correspond to proteins enriched in FF-treated sperm compared with Controls. Functional clusters were annotated with representative biological processes, including galactose metabolism and zona pellucida binding. **(D)** Additional STRING subnetwork of proteins underabundant in FF relative to Control, highlighting clusters related to endoplasmic reticulum chaperone complexes and Golgi lumen acidification. White-to-red halo intensity indicates the magnitude of decreased abundance in FF-treated sperm.

To evaluate the effect of follicular fluid on the proteome of capacitated alpaca spermatozoa, we compared samples incubated in Fert-TALP supplemented with 20% FF to those in Fert-TALP alone (Control). The volcano plot (Figure 4A; Supplementary Table 1) showed a clear proteomic shift, with 33 proteins significantly more abundant and 13 less abundant in the FF group (|FC| ≥ 2, *p* < 0.05). Underrepresented proteins were mainly associated with the endoplasmic reticulum and Golgi apparatus, including ERp44, ATP6AP1/2, CPD, SMPDL3A, ZG16, and TCN2 (Figure 4D). In contrast, proteins enriched in FF-treated sperm formed a highly interconnected network (Figure 4C). The largest cluster (12 nodes) included proteins linked to sperm–zona pellucida binding (e.g., LYPD4, PGK1, ANXA2, TCP1 complex components) and galactose metabolism (e.g., MAOA, AKR1B1, GLA, HK1). GO enrichment analysis (Figure 4B) further highlighted processes consistent with the functional improvements in FF-treated sperm, such as glycolysis/gluconeogenesis, actin cytoskeleton organization, protein maturation, and interleukin signaling.

### 3.5 Oviductal fluid downregulates capacitation-associated proteins in alpaca spermatozoa

**Figure 5:**
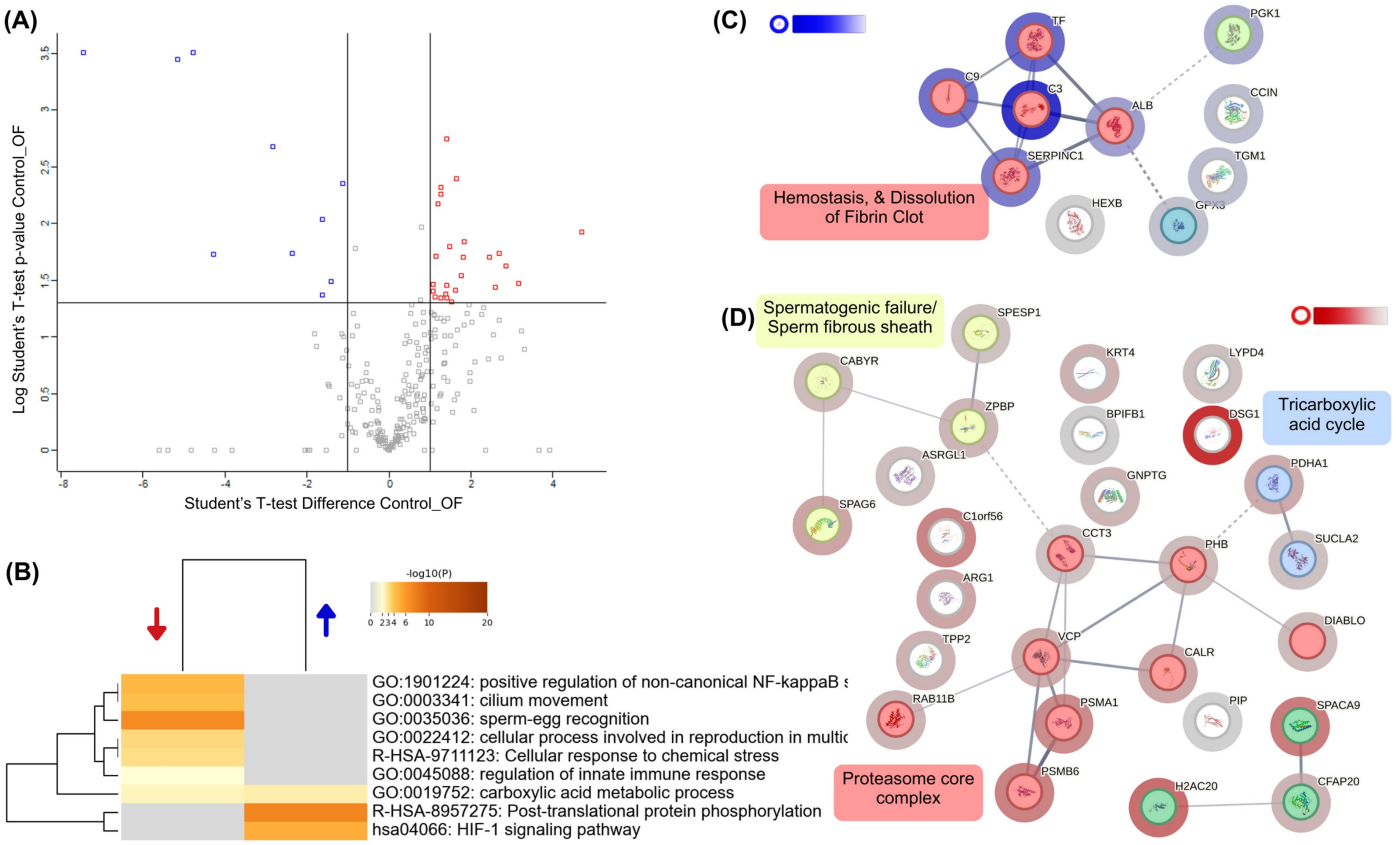
Differential proteomic profile of alpaca spermatozoa incubated with oviductal fluid (OF). **(A)** Volcano plot showing proteins differentially abundant between Control (Fert-TALP) and OF-treated spermatozoa. Blue dots indicate proteins overabundant in OF-treated sperm, while red dots represent proteins underabundant (|FC| ≥ 2, *p* < 0.05). **(B)** Heatmap of enriched biological processes identified by Metascape. Blue and red arrows indicate processes associated with overabundant and underabundant proteins, respectively. Color intensity corresponds to –log₁₀(*p*-value). **(C)** Protein–protein interaction (PPI) network generated by STRING, clustered using k-means. Nodes are colored according to functional clusters, with halo intensity reflecting the degree of differential abundance: blue halos correspond to proteins enriched in OF-treated sperm. Clusters were functionally annotated, including processes such as hemostasis and fibrin clot dissolution. **(D)** Additional STRING subnetwork highlighting proteins underabundant in OF relative to Controls, with clusters associated with spermatogenic defects/fibrous sheath integrity, the tricarboxylic acid (TCA) cycle, proteasome core complex, and ER stress response. White-to-red halo intensity indicates the magnitude of decreased abundance in OF-treated sperm.

To assess the impact of oviductal fluid on capacitated alpaca sperm, we compared samples incubated in Fert-TALP with 20% OF to Control. The volcano plot (Figure 5A; Supplementary Table 1) revealed only 11 proteins overabundant and 29 underabundant in OF (|FC| ≥ 2, *p* < 0.05). Many downregulated proteins were linked to sperm function (CFAP20, SPACA9, CABYR, and SPAG6), flagellum structure, fibrous sheath motility, and sperm–egg interaction. STRING analysis (Figure 5D) identified three downregulated clusters: proteasome-related proteins, motility/structural proteins, and TCA cycle components. GO enrichment (Figure 5B) highlighted diminished processes such as cilium movement, sperm–egg recognition, and stress responses, whereas proteins enriched in OF were mainly associated with general pathways such as HIF-1 signaling and protein phosphorylation”, as well as a seven-protein cluster related to hemostasis and fibrin clot dissolution (Figure 5C).

### 3.6 Follicular fluid induces broader and functionally integrated proteomic remodeling compared with oviductal fluid

**Figure 6:**
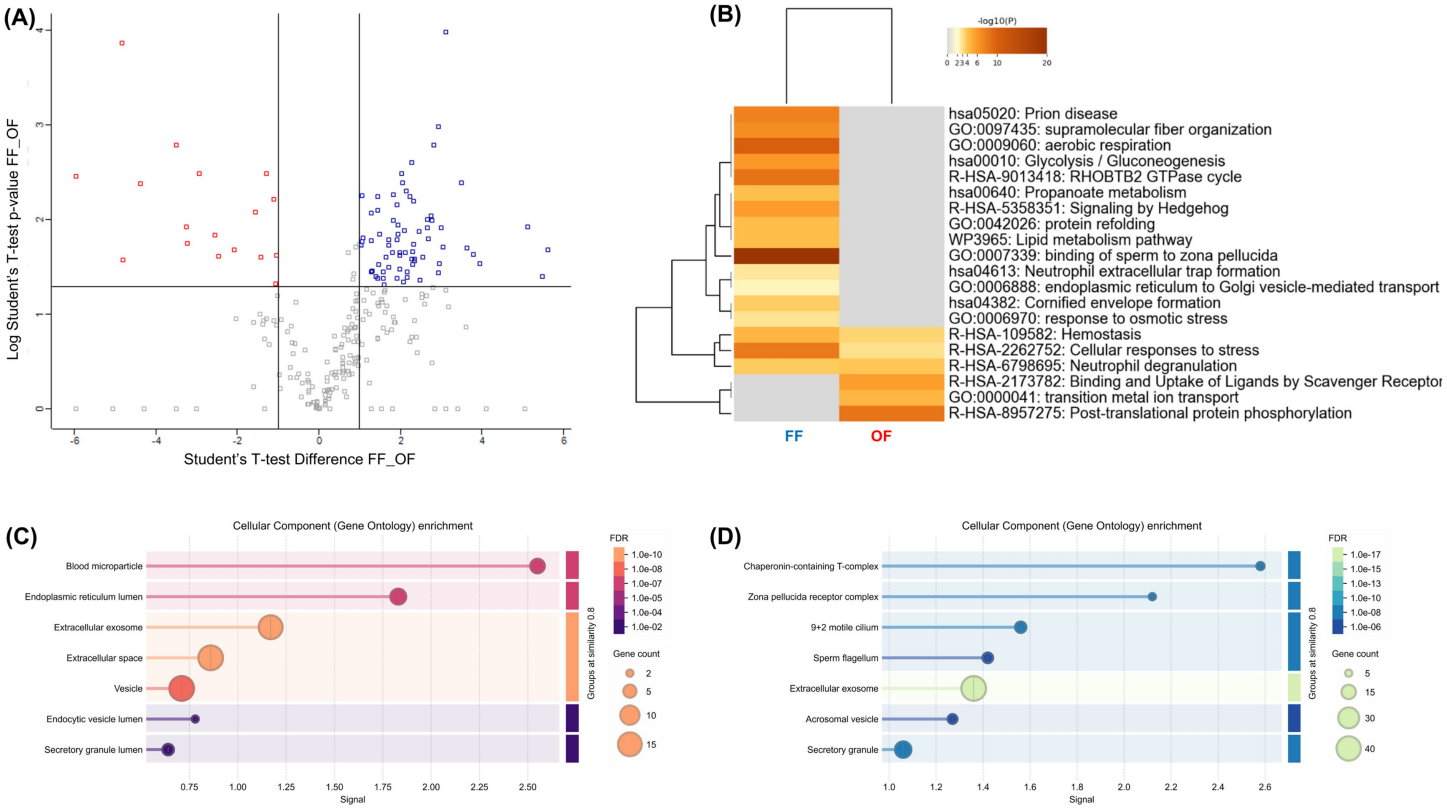
Comparative proteomic analysis of alpaca spermatozoa incubated with follicular fluid (FF) or oviductal fluid (OF). **(A)** Volcano plot displaying differentially abundant proteins between FF- and OF-treated spermatozoa (|FC| ≥ 2, *p* < 0.05). Blue dots represent more abundant proteins in the FF group, while red dots indicate proteins enriched in the OF group. **(B)** Heatmap of functional enrichment (Metascape). Color intensity corresponds to –log₁₀(*p*-value). **(C,D)** Bar plots of Gene Ontology enrichment. **(C)** Enriched processes in OF-treated spermatozoa (red-to-orange scale, adjusted FDR shown on right). **(D)** Enriched processes in FF-treated spermatozoa (blue-to-green scale). Circle size represents gene count associated with each term.

To directly compare the effects of FF and OF, a differential proteomic analysis was performed. The volcano plot (Figure 6A; Supplementary Table 1) revealed a strong asymmetry, with 76 proteins enriched in FF-treated sperm *versus* 17 in the OF group (|FC| ≥ 2, *p* < 0.05). Functional enrichment (Figures 6B–D) showed that FF supplementation favored pathways critical for fertilization, including binding of sperm to zona pellucida (GO:0007339), aerobic respiration (GO:0009060), glycolysis/gluconeogenesis (hsa00010), lipid metabolism (WP3965), and response to osmotic stress (GO:0006970). FF-enriched proteins also mapped to key cell compartments such as the flagellum, acrosomal vesicle, and zona pellucida receptor complex. In contrast, OF-treated sperm showed enrichment in more general processes, including post-translational protein phosphorylation (R-HSA-8957275) and ligand uptake by scavenger receptors (R-HSA-2173782), associated mainly with the endoplasmic reticulum and extracellular vesicles. These results reinforce that FF drives a broader and functionally coordinated remodeling of the sperm proteome compared with OF.

### 3.7 Comparative proteomic signatures of follicular and oviductal fluids and their overlap with capacitated spermatozoa

**Figure 7:**
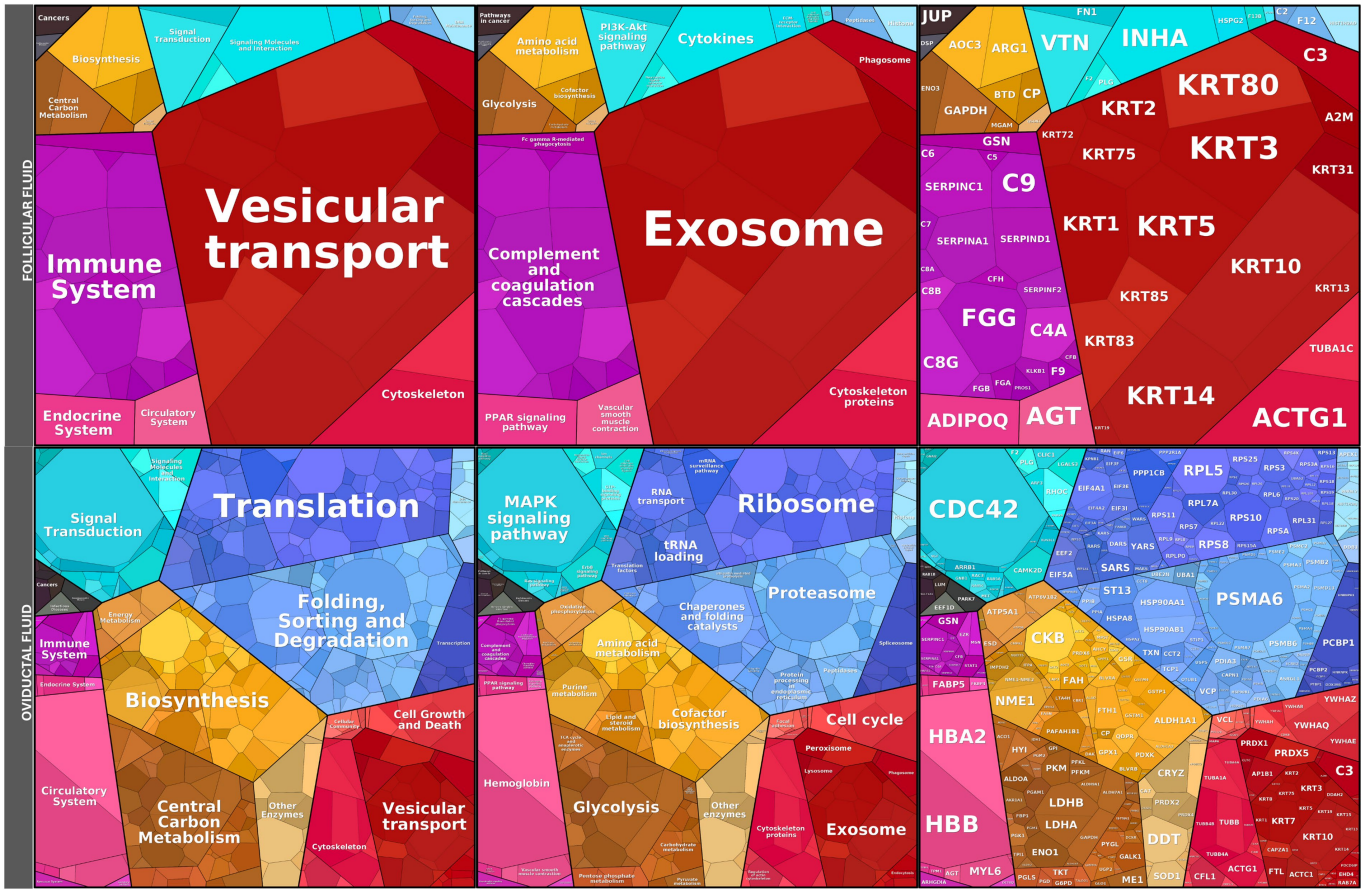
Proteomaps of follicular fluid (FF) and oviductal fluid (OF) proteomes. Functional landscape of proteins identified in FF and OF. Rectangles represent proteins grouped into functional categories based on KEGG pathways, where the size of each polygon reflects the relative abundance of the corresponding protein. FF proteome (top) was enriched in categories related to vesicular transport, exosomes, immune system regulation, and complement/coagulation cascades, consistent with roles in intercellular communication and modulation of sperm membrane remodeling. In contrast, the OF proteome (bottom) displayed predominant enrichment in translation, ribosome, proteasome, MAPK signaling, and biosynthesis pathways, reflecting a more general representation of cellular housekeeping and protein-folding machinery. Together, these data illustrate the distinct molecular composition of peri-ovulatory fluids in alpacas, suggesting specialized contributions of FF and OF to sperm function.

The proteomic landscape of FF and OF was further explored using Proteomaps (Figure 7; Supplementary Tables 2, 3). In FF, dominant categories included vesicular transport, exosome-associated proteins, and complement/coagulation cascades, together with contributions from metabolic and immune pathways. These features are consistent with the role of FF as a source of extracellular vesicles and signaling proteins that interact with spermatozoa to facilitate membrane remodeling, capacitation, and acrosome priming. By contrast, the OF proteome displayed a markedly different profile, enriched in translation, ribosomal, and proteasomal proteins, as well as MAPK signaling, biosynthesis, and glycolysis-related pathways. The predominance of protein synthesis and quality-control machinery in OF suggests a role in maintaining oviductal epithelial homeostasis and supporting sperm preservation rather than directly promoting capacitation. Collectively, these findings highlight that FF provides a more specialized proteomic environment geared toward modulating sperm function, whereas OF reflects a broader cellular maintenance profile.

Comparison of fluid proteomes with spermatozoa capacitated in the presence of FF or OF revealed limited overlap for FF and broader intersections for OF (Supplementary Figure 1). In FF (Supplementary Figure 1A), only ANXA2 and QSOX1 were shared with overabundant sperm proteins, suggesting roles in membrane remodeling and redox balance, while no overlap was observed with underabundant proteins. In OF (Supplementary Figure 1B), seven proteins (LDHB, C9, SERPINC1, ALB, PGK1, C3, TF) overlapped with overabundant sperm proteins, mainly related to metabolism and immune regulation, and eight proteins (e.g., CALR, CCT3, VCP) were shared with underabundant sperm proteins, associated with protein folding and remodeling.

## 4 Discussion

Artificial insemination (AI) in alpacas and llamas continues to be characterized by low pregnancy rates, typically ranging between 10 and 30% under field conditions, and in some cases dropping below 10% with the use of frozen-thawed semen (1, 17, 35, 36). These limitations are largely associated with the high viscosity of seminal plasma, decreased sperm survival following processing, and an incomplete understanding of the molecular mechanisms governing sperm capacitation in camelids (36, 37). Therefore, improving capacitation media efficiency is a key prerequisite for enhancing AI success in this species.

In this context, this study provides new insights into the influence of FF and OF on the functional and proteomic landscape of alpaca spermatozoa, contributing to the identification of physiological factors that may enhance *in vitro* capacitation and fertilization competence. Supplementation of the capacitation medium with 20% FF significantly enhanced sperm quality parameters compared with both the Control and OF treatments. Specifically, FF increased progressive motility (↑72.1%) and rapid progressive motility (↑169%), as well as curvilinear velocity (↑7.2%) and curvilinear distance (↑23.9%), reflecting a more vigorous and extensive motility pattern consistent with hyperactivation, as reported in other species (38). This functional improvement was accompanied by higher viability (↑30%) and a greater proportion of reacted acrosomes (↑30%), suggesting that FF promotes a capacitation-associated motility state favorable for fertilization.

In alpacas, the evaluation of plasma membrane integrity after capacitation showed no significant alterations following FF or OF supplementation, with membrane integrity remaining above 40% and improvements in sperm kinetic parameters. These findings reinforce that maintaining a functional membrane is essential for successful sperm capacitation in SAC. Moreover, Sofikitis (39) highlighted that motility, membrane integrity, cellular functionality, and DNA integrity are closely related to the sperm’s ability to activate and fuse with the ooplasm of the human oocyte. These factors are critical for the success of *in vitro* fertilization, underscoring the importance of preserving membrane functionality as a core requirement for capacitation.

Hypermotility is a hallmark of flagellar hyperactivation, which physiologically occurs when spermatozoa are released from the oviductal reservoir after exposure to peri-ovulatory FF (9, 40). Consistent with this, it was demonstrated that FF promotes epididymal sperm capacitation and acrosome reaction in alpacas, accompanied by increased curvilinear velocity and amplitude of lateral head displacement (41). Our findings extend these observations to ejaculated spermatozoa, confirming that FF provides conserved bioactive components capable of stimulating capacitation-like changes. Similar results have been reported in rams, where FF supplementation improved motility and viability (7), underscoring that FF across species contains conserved capacitation-promoting molecules, including cholesterol acceptors, annexins, and growth factors.

The positive effects of FF are concentration-dependent: low-to-moderate concentrations (5–30%) have proven beneficial in rams and cattle (7, 17), whereas higher concentrations (50–100%) impair motility and viability (1, 42). In this study, supplementation with 20% FF (0.29 mg protein/100 μL medium) maintained viability above 50% and increased total motility (~60%), supporting the suitability of this concentration for camelid sperm capacitation.

Our proteomic analyses provide mechanistic support for the functional improvements observed in FF-treated spermatozoa. Several proteins significantly enriched under FF supplementation are directly linked to energy metabolism and capacitation. Components of the mitochondrial ATP synthase complex (ATP5F1A, ATP5F1B, ATP5PB, ATP6V1E2) and the TCA cycle (SUCLA2), all key factors of the proton-transporting two-sector ATPase complex and ATP formation by chemiosmotic coupling, were increased in FF-treated sperm relative to OF treatment. This metabolic reinforcement is consistent with the enhanced motility and hyperactivation observed, as mitochondrial ATP production is essential for vigorous flagellar activity in boar (43, 44). Similar findings have been reported in capacitated ram spermatozoa, where energy production and conversion enzymes (ATP6V1H, ATP6V1F, ATP5PD, and ATP5IF) were also differentially enriched (45).

Similarly, the overabundance of antioxidant enzymes such as PRDX5 and AKR1B1 in FF-capacitated sperm suggests a protective role of FF in controlling ROS levels, preventing lipid peroxidation, and enabling ROS-dependent tyrosine phosphorylation cascades essential for capacitation in humans (46–48). PRDX5 was also differentially expressed in boar sperm during capacitation, where it may support post-capacitation processes, particularly acrosome exocytosis and sperm–egg binding (43, 44), and it has recently been detected in llama sperm (49).

Kwon (50) reported overexpression of proteins belonging to the sperm–egg interaction ontology group during boar sperm capacitation. In alpacas, we similarly observed that proteins related to sperm–egg interaction were highly represented in the FF group. ZPBP (zona pellucida–binding protein), SPACA9, and SPACA6 are acrosome-associated proteins critical for sperm binding and penetration of the zona pellucida, previously described in men, mice, and boars (51). Moreover, Park (52) showed that SPACA1 and SPACA5 were newly synthesized in the heads of normal-fertility bull spermatozoa. Together with vesicular transport regulators such as RAB2A and RAB11B, which were overabundant in the FF group, these findings point to an enhanced readiness of the acrosomal machinery and membrane remodeling processes, in line with the increased acrosome reaction reported in boars (44, 53).

Importantly, several proteasome subunits (PSMA1, PSMB6, PSMG6, PSMA8) were overabundant in FF-treated sperm, whereas the proteasome core complex was reduced in OF-treated sperm. Interestingly, PSMA6 and PSMA8 were also identified in ejaculated llama sperm (49). The proteasome system plays a central role in regulating sperm capacitation, acrosome reaction, and zona pellucida penetration by degrading zona proteins and modulating exocytotic events in boar (54, 55).

In addition, the Proteomap of alpaca FF revealed enrichment in vesicular transport and exosome-related proteins. Exosomes and other extracellular vesicles derived from FF are recognized as carriers of proteins, lipids, and signaling molecules that contribute to sperm capacitation, acrosome reaction, and fertilization by transferring bioactive molecules to the sperm membrane (56, 57). In our study, this was reflected in the enrichment of annexins, apolipoproteins, and stress-response chaperones in FF, which may act as mediators of membrane remodeling and acrosomal priming. Moreover, ANXA2 and QSOX1 were detected in FF and found to be overabundant in FF-treated sperm, underscoring their potential relevance to capacitation. ANXA2 has also been reported in llama sperm (Sari et al., 2023) and is known to participate in membrane remodeling during capacitation in humans, boars, and mice (58). Notably, in humans, ANXA2 is typically detected only in sperm of excellent quality, whereas its under-expression has been associated with infertility. This suggests that ANXA2 may contribute to the structural and functional changes that spermatozoa undergo within the oviduct (58), possibly through delivery by exosomes (59). QSOX1, a recognized component of human exosomes (60), has been implicated in improving sperm quality in human and mouse semen by protecting cells against oxidative stress and preserving structural integrity (61, 62). Together, these findings support a role for ANXA2 and QSOX1 in enhancing sperm resilience and functionality during capacitation, consistent with their detection in FF and FF-treated alpaca sperm.

Beyond the roles of ANXA2 and QSOX1, which underscore the influence of FF-derived proteins on sperm capacitation, it is vital to assess how synthetic formulations differ from natural reproductive fluids. Synthetic oviduct fluid (SOF), although prevalent in cattle, sheep, and goats, lacks protein and thus requires fortification with BSA, hormones, or serum to support capacitation and embryo development (21, 63–65). Comparative studies show that while SOF sustains cleavage, blastocyst quality, or hatching, it often fails to replicate the capacitation-promoting effects of native OF (21, 63). Our results suggest that proteins such as ANXA2 and QSOX1, enriched in alpaca FF and FF-treated sperm, could be promising supplements. Their addition to base media such as Fert-TALP or SOF may advance camelid sperm capacitation by enhancing membrane remodeling, mitigating oxidative stress, and promoting acrosomal preparedness. Therefore, future research comparing natural OF with protein-supplemented synthetic media will be crucial for developing alpaca-specific media that more accurately mimic the reproductive tract environment and enhance ART outcomes.

In cattle, OF proteins have been shown to modulate sperm viability and motility (66–68). Mahé (69) reported that incubating sperm with 0.36 mg/100 μL OF for 3 h, the same concentration and incubation time used in this study, resulted in increased sperm membrane fluidity when the fluid originated from the post-ovulatory ampulla, while OF from the post-ovulatory isthmus preserved higher sperm viability. In contrast, pre-ovulatory ampulla fluid exhibited stronger protein interactions with sperm. In alpacas, however, OF-treated spermatozoa did not display any functional enhancement of motility, kinetics, viability, or acrosome reaction relative to the Control group and instead showed detrimental effects compared with FF. Proteomic analysis further revealed a downregulation of capacitation-related processes. Most of the relevant proteins were under-expressed in the OF group, including structural and motility-associated proteins such as SPAG6, CABYR, CFAP20, and SPACA9, whose reduced abundance is consistent with impaired fibrous sheath integrity and diminished capacity for hyperactivation. Pathway analyses indicated decreased representation of proteasome-related proteins, TCA cycle components, and cilium movement—processes essential for capacitation, acrosomal remodeling, and zona pellucida penetration in boars (50, 55). OVGP1, known to promote sperm viability, motility, and capacitation in bulls (69), was identified in the alpaca OF proteome. However, the fluid also contained PEBP1 and PAFAH1B3. These proteins are decapacitation factors that stabilize sperm membranes and delay acrosomal responsiveness in mice and humans (70, 71). This dual presence highlights the complexity of OF and suggests a stage-dependent role, supporting sperm survival while restraining premature capacitation.

These cross-species comparisons highlight mechanisms conserved in sperm capacitation, including energy metabolism, proteasome activity, and acrosomal remodeling. They also highlight camelid-specific traits, like the prevalence of decapacitation factors in OF and the enrichment of exosome-related proteins in FF. This context situates alpaca sperm physiology within mammalian reproduction, while emphasizing unique adaptations in SAC.

This study is strengthened by its robust design: ejaculates from three individuals (five per individual) were analyzed, with independent functional assays (*n* = 5 per individual) and pooled samples run in triplicate for proteomics. FF and OF were collected at the pre-ovulatory stage from 7 to 9 mm follicles, representing the physiological window of sperm deposition in the female tract of camelids during mating. Although proteomic coverage was moderate (≈650 proteins in OF, ≈100 in FF, ≈250 in sperm), the data provided biologically meaningful, stage-specific insights consistent with the functional results. Limitations include the use of whole pre-ovulatory OF, which may have restricted sperm responses. Moreover, obtaining early post-ovulatory OF from slaughterhouse samples is nearly impossible, restricting direct comparisons with cattle. Future studies should refine OF analysis by separating ampulla and isthmus fractions, incorporating both pre- and post-ovulatory stages, and testing variable incubation times and concentrations. In addition, because *Vicugna pacos* is absent from current proteomic annotation platforms, functional enrichment relied on mapping to human orthologs, a well-established approach in livestock proteomics that enables pathway analysis and comparative insights, while acknowledging that species-specific mechanisms may not be fully captured.

## 5 Conclusion

This study is the first to provide a partial proteome of capacitated alpaca spermatozoa, along with analyses of follicular and oviductal fluids. In combination with our functional data, these findings highlight the complementary but contrasting roles of follicular and oviductal fluids in modulating alpaca sperm physiology. Follicular fluid promoted a coordinated proteomic response that enhanced motility vigor, hyperactivation, antioxidant defense, and zona pellucida–binding capacity, reflecting its physiological role in triggering capacitation at ovulation. In contrast, oviductal fluid induced a proteomic profile enriched in decapacitation factors and membrane-stabilizing proteins, consistent with its role in preserving sperm within the oviductal reservoir until the appropriate peri-ovulatory signals arise. These findings emphasize the fluid-specific molecular cues and species-specific reproductive strategies governing sperm function and suggest that follicular fluid may represent a promising supplement for optimizing *in vitro* capacitation protocols in camelids, thereby contributing to improved assisted reproduction outcomes.

## Data Availability

The original contributions presented in the study are publicly available. The mass spectrometry proteomics data have been deposited to the ProteomeXchange Consortium via the PRIDE partner repository with the dataset identifier PXD068210.

## Supplementary material

SUPPLEMENTARY FIGURE 1 Venn diagrams comparing follicular fluid (FF) and oviductal fluid (OF) proteomes with sperm proteomes after treatment. **(A)** Comparison of FF proteins with sperm treated with FF proteins that were overabundant (S-FF [+]) or underabundant (S-FF [−]) relative to control. Only two proteins, ANXA2 and QSOX1, were shared between FF and S-FF (+). (B) Comparison of OF proteins with sperm treated with OF proteins overabundant (S-OF [+]) or underabundant (S-OF [−]) relative to control. Seven proteins were shared with S-OF (+), while eight proteins overlapped with S-OF (−). Percentages indicate the proportion of total proteins in each group.

SUPPLEMENTARY TABLE 1 Differential proteomic profile of alpaca spermatozoa incubated with follicular fluid (FF) or oviductal fluid (OF). Normalized protein abundance values obtained from spermatozoa incubated under three conditions: control medium (Fert-TALP), Fert-TALP supplemented with 20% FF, and Fert-TALP supplemented with 20% OF. The table includes UniProt accession, protein description, gene name, normalized abundance values, fold-change differences between groups, and Student’s *t*-test *p*-values. These data provide the basis for the volcano plots, protein–protein interaction analyses, and functional enrichment presented in the main manuscript.

SUPPLEMENTARY TABLE 2 Proteomic profile of alpaca follicular fluid (FF).List of proteins identified in FF samples (*n* = 20, pooled from pre-ovulatory follicles 7–9 mm), including UniProt accession, description, gene names, molecular weight (MW), theoretical isoelectric point (pI), peptide counts, peptide-spectrum matches (PSMs), Sequest HT scores, and relative protein abundance expressed as emPAI%.

SUPPLEMENTARY TABLE 3 Proteomic profile of alpaca oviductal fluid (OF). List of proteins identified in OF samples (*n* = 10, pooled from pre-ovulatory follicles;7 mm), including UniProt accession, description, gene names, molecular weight (MW), theoretical isoelectric point (pI), peptide counts, peptide-spectrum matches (PSMs), Sequest HT scores, and relative protein abundance expressed as emPAI%.
