# Supplementary figures and images for: Proteomic and functional analysis of alpaca (Vicugna pacos) sperm quality following in vitro capacitation with follicular and oviductal fluids
Source: Front Vet Sci. 2025 Nov 6;12:1702095. doi: 10.3389/fvets.2025.1702095 (PMC12631973; doi:10.3389/fvets.2025.1702095)

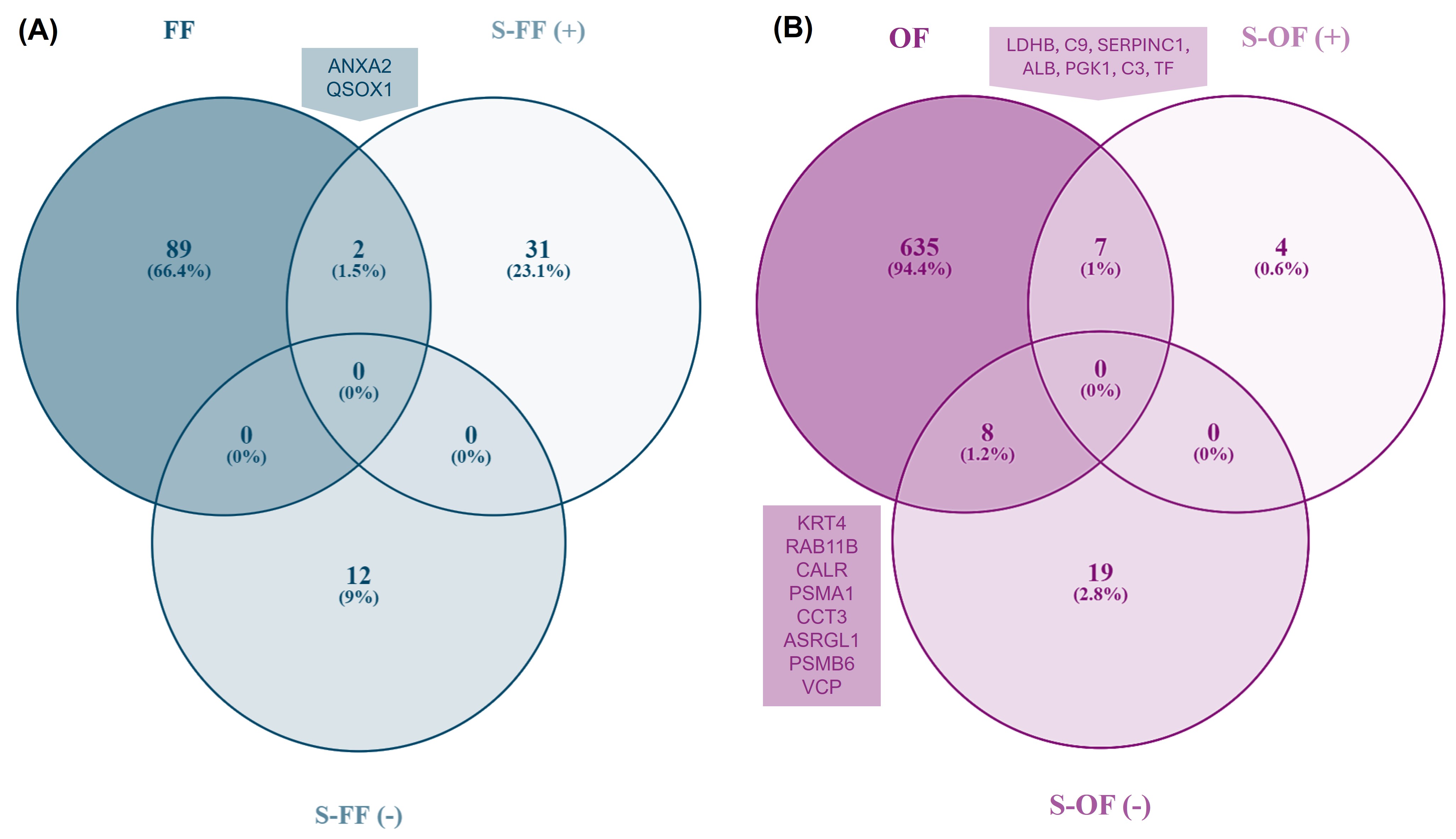

Supplement: SUPPLEMENTARY FIGURE 1 — Venn diagrams comparing follicular fluid (FF) and oviductal fluid (OF) proteomes with sperm proteomes after treatment. (A) Comparison of FF proteins with sperm treated with FF proteins that were overabundant (S-FF [+]) or underabundant (S-FF [−]) relative to control. Only two proteins, ANXA2 and QSOX1, were shared between FF and S-FF (+). (B) Comparison of OF proteins with sperm treated with OF proteins overabundant (S-OF [+]) or underabundant (S-OF [−]) relative to control. Seven proteins were shared with S-OF (+), while eight proteins overlapped with S-OF (−). Percentages indicate the proportion of total proteins in each group. [file Image_1.jpeg]
